# Functional nanostructures for electronics, spintronics and sensors

**DOI:** 10.3762/bjnano.11.152

**Published:** 2020-11-10

**Authors:** Anatolie S Sidorenko

**Affiliations:** 1 D. Ghitu Institute of Electronic Engineering and Nanotechnologies, Chisinau, Moldova and Orel State University, Orel, Russia

**Keywords:** functional nanostructures, nanoelectronics, post-Moore generation, sensors, spintronics, supercomputers

Nanotechnology and functional nanostructures, exciting trends of the 21st century, are topics that have penetrated and influenced nearly all areas of human activity: from microelectronics to biology, from aerospace to medicine, from agriculture to novel materials engineering. One of the areas of nanoscience and nanotechnology that is developing especially rapidly is research on functional nanostructures for targeted applications. One of the most important and promising of these targeted applications are superconducting spintronic nanostructures for supercomputers of novel, “post-Moore” generation.

The exponential development of microelectronics and computers, based on traditional semiconductor chips, has followed the empirical Moore’s Law (formulated by one of the founders of Intel Corporation, Gordon Moore) for over four decades. Since 1965, Intel has designed chips that fit twice as many transistors into the same space of the chip every two years, following an exponential curve – this size shrinking of permanent transistors has made computers more powerful and compact. Nevertheless, in the last decade, a clear deviation has appeared – a slowing of Moore’s Law. Bob Colwell, the architect on the Pentium Pro, Pentium II, Pentium III and Pentium IV, described the stagnation of semiconductor technology as follows [1]:

“Officially Moore’s Law ends in 2020 at 7 nm, but nobody cares, because 11 nm isn’t any better than 14 nm, which was only marginally better than 22 nm … thermal dissipation issues thoroughly constraining the integration density, the multicore era effectively ends, leading to the “dark silicon” problem, i.e., only parts of available cores can be run simultaneously”.

Energy efficiency has now become a crucial parameter, limiting the advancement of supercomputers. The powerful, modern supercomputer, the Sunway Taihu Light [2], with a peak performance of 93 peta FLOPS (93 × 10^15^ floating point operations per second) has an energy consumption as high as 15.4 MW. This corresponds to a power plant capacity able to supply energy to a middle-sized city!

The low energy efficiency leads to high power consumption, and at the same time, limits the clock frequency of semiconductor computers to 4–5 GHz. This frequency limit occurs due to temperature limitations posed at the integration level and the switching rate of transistors.

It is important to realize that cryogenic cooling of semiconductor chips will not solve the problem [3]. The future of high-performance computing will most likely be associated with one of the alternative “post-Moore’s” technologies where energy dissipation is drastically lower. It is expected that the most promising “post-Moore’s” candidate to lead the technological way is superconductor digital technology (SDT) [4]. The switching energy of the SDT basic element is on the order of 10^−19^ J (including the power for cryogenic cooling of superconducting circuits), which demonstrates an energy efficiency of up to seven orders of magnitude as compared to the semiconductor analog [5]. The competitiveness of SDT illustrates a working prototype for a superconducting computer developed under the “Cryogenic computing complexity” IARPA program [6]. This is a 64-bit computing machine operating at a 10 GHz clock frequency with a throughput of 10^13^ bit-op/s and an energy efficiency of 10^15^ bit-op/J at a temperature of 4 K. A prospective investigation found that a superconductor computer could outperform its semiconductor competitor by two orders of magnitude in energy efficiency, demonstrating 250 GFLOPS/W [7]. The base elements of the superconductor computer are superconductor logic and memory circuits, where some of these prospective examples and the technological processes related to their fabrication are presented in this thematic issue.

In the last decade, very rapid development in a subfield of solid-state physics and engineering – superconducting spintronics based on functional nanostructures consisting of alternating layers of ferromagnetic and superconducting materials – has been observed. Due to the proximity effect of superconductor/ferromagnetic (S/F) layers and Andreev reflection of Cooper pairs at the S/F interface, a number of new phenomena were first theoretically predicted and then experimentally detected. Some examples include a nonuniform superconducting Fulde–Ferrell–Larkin–Ovchinnikov (FFLO) state, S/F π-junctions, oscillations of critical temperature and critical current in S/F hybrids on the thickness of the F-layer, multiperiodic re-entrant superconductivity, triplet pairing and triplet spin-valve and memory effects – just to name some of the new phenomena that have been detected in layered S/F hybrid nanostructures [8]. Moreover, the detected effects are very promising for technical applications directed towards enhancing the storage capacity of computer memory and the potential use as quantum computer building blocks.

Keeping in mind the trends with regard to “post-Moore’s” electronics, this thematic issue aimed to cover theoretical, experimental and conceptual development towards superconducting supercomputer elements. Theoretical works on Josephson junctions as base elements for a superconducting computer were presented by Karabassov et al. [9] and Marychev et al. [10]. In the latter, the authors present a theoretical study of the current–phase relation of very promising SN-S-SN Josephson junctions, which could serve as energy efficient, high-performance superconducting electronics elements for fast computing.

This issue also contains progress towards various technological processes for fabrication and characterization of the base elements of a superconducting computer. For example, Arutyunov et al. [11] presented an advanced technology including lift-off electron-beam lithography followed by ultra-high-vacuum deposition of materials that was used for fabrication of nanostructured quasi-1D chains of Josephson junctions. This was followed by the work of Mohammed et al. [12] who presented a smart vacuum technology for the design of hetero-epitaxial S/F nanostructures. The elaborated and fabricated nanostructures can be utilized in superconducting memory and logic circuits as Josephson magnetic random access memory (MRAM) elements for a superconducting computer.

Other articles, more conceptual in nature, where the authors proposed, calculated, fabricated and investigated the novel base elements and electronic circuits for a superconducting computer include work by Bakurskiy et al. [13], where a tunable kinetic inductor is proposed as an artificial synapse for a superconducting neuronal network. This work combines the results of theoretical and experimental investigations of S/F superlattices. Such superlattices can be used as tunable kinetic inductivity synapses in artificial neural networks of a superconducting computer with non-von Neumann architecture. A further example by Novikov et al. [14] demonstrated the concept of “read-out” electronics for a superconducting computer where a low-noise cryogenic microwave amplifier as a read-out circuit of superconducting X-mon qubits is demonstrated.

Also, in addition to the progress towards theoretical and experimental developments of a superconducting computer, this thematic issues also includes several articles devoted to extra-sensitive detectors, their theoretical basis and technological process in terms of fabrication. For example, a novel phenomenon was presented in [15], which predicted the phenomena of superconductor–insulator quantum phase transitions in ultrathin capacitively coupled superconducting nanowires with quantum phase slips which may be used for interpretation of already existing experiments on meander-like nanowires and for the design of a novel set of superconducting sensors. Another very promising photon detector [16] was demonstrated for supersensitive detection in astrophysics and read-out tracts measuring signals generated by quantum circuits at a frequency of 6–9 GHz.

Summarizing the above-mentioned milestones, one can see that the main focus of this thematic issue is the new area of research: superconductor/ferromagnetic hybrid nanostructures and their applications for quantum electronics and spintronics. In addition to these highlighted works, there are also other interesting functional nanostructures, sensors and quantum detectors presented, to highlight the fascinating world of nanoelectronics.

The concept of this thematic issue emerged during the international SPINTECH conference “NANO-2019: Limits of Nanoscience and Nanotechnologies” and the summer school it followed, “S/F Hybrid Structures for Spintronics”, that took place in September 2019 in Chisinau, Moldova. Presented by many of the participants of the conference and lecturers of the summer school, new ideas, technological approaches to design of functional nanostructures for superconducting spintronics, quantum electronics, sensors and novel base elements for superconducting supercomputers are the core of this thematic issue.

As the thematic issue editor, I would like to thank all highly experienced experts from 15 countries who presented novel, original results at the NANO-2019 conference and those who submitted valuable contributions to this issue. We believe that this thematic issue will attract the attention of scientists, technologists, and engineers and will be useful for a broad readership. The professional and permanent kind editorial support by the Production Team of the Beilstein Journals is greatly acknowledged. A.S. also thanks the SPINTECH project (G.A. Nr. 810144) and the Grant RSF Nr. 20-62-47009 “Physical and engineering basis of computers non-von Neumann architecture based on superconducting spintronics” for the support.

Anatolie Sidorenko

Chisinau, July 2020
